# Energy Optimization Using a Case-Based Reasoning Strategy

**DOI:** 10.3390/s18030865

**Published:** 2018-03-15

**Authors:** Alfonso González-Briones, Javier Prieto, Fernando De La Prieta, Enrique Herrera-Viedma, Juan M. Corchado

**Affiliations:** 1 BISITE Digital Innovation Hub, University of Salamanca, Edificio I+D+I, 37007 Salamanca, Spain; javierp@usal.es (J.P.); fer@usal.es (F.D.L.P.); 2 Department of Computer Science and Artificial Intelligence, University of Granada, 18071 Granada, Spain; 3 Department of Electronics, Information and Communication, Faculty of Engineering, Osaka Institute of Technology, Osaka 535-8585, Japan; 4 Pusat Komputeran dan Informatik, Universiti Malaysia Kelantan, Karung Berkunci 36, Pengkaan Chepa, Kota Bharu 16100, Kelantan, Malaysia

**Keywords:** smart building, ubiquitous computing, intelligent management, case-based reasoning

## Abstract

At present, the domotization of homes and public buildings is becoming increasingly popular. Domotization is most commonly applied to the field of energy management, since it gives the possibility of managing the consumption of the devices connected to the electric network, the way in which the users interact with these devices, as well as other external factors that influence consumption. In buildings, Heating, Ventilation and Air Conditioning (HVAC) systems have the highest consumption rates. The systems proposed so far have not succeeded in optimizing the energy consumption associated with a HVAC system because they do not monitor all the variables involved in electricity consumption. For this reason, this article presents an agent approach that benefits from the advantages provided by a Multi-Agent architecture (MAS) deployed in a Cloud environment with a wireless sensor network (WSN) in order to achieve energy savings. The agents of the MAS learn social behavior thanks to the collection of data and the use of an artificial neural network (ANN). The proposed system has been assessed in an office building achieving an average energy savings of 41% in the experimental group offices.

## 1. Introduction

From the beginning of the nineteenth century, social and economic development among sophisticated societies was triggered by the large-scale use of fossil fuels. Petroleum and coal were the main sources of energy, which not only have been burned in excessive amounts but also over a long period of time. This has had long term negative effects on our environment such as contamination and climate warming caused by the harmful gases released into the atmosphere and dangerous waste spills. Given these negative impacts and the fact that these resources are becoming scarce, it is necessary to find solutions that will make countries less reliant on fossil fuels. The main advantage of renewable energies is that their use does not imply as many negative consequences for our environment, as they reduce the amount of pollutants released into the atmosphere. Thus, we must be firmly committed to using renewable and clean sources of energy. In addition, their territorial distribution is more disperse. The EU launched an integrated policy for energy and climate change. Its aim is to move Europe towards a sustainable future and an economy that produces low carbon emissions and consumes less energy [1]. 

This new paradigm also affects the generation of electrical energy, where competition increased after a decentralized approach has been adopted. In many countries, this model is already beginning to capture part of the revenues of conventional business, undermining its benefits and adding complexity to the already difficult task of balancing supply and demand. Overcapacity, low prices in the electricity market for increased renewable production and a reform that penalizes investment in clean energy resources, add to this difficulty. Meanwhile, the European Commission has set very ambitious green energy targets to be achieved by 2030, such as the use of 27% of clean resources, 36% less CO_2_ emissions and a 27% increase in energy efficiency [2,3].

Proposals focusing on energy efficiency that we found in the state of the art have numerous weaknesses. The work of Zhao et al., proposes the use of a multi-agent architecture for decision-making in energy system management [4]. However, this system lacks knowledge on the environment and thus cannot make appropriate decisions; this is because it does not use external sources to incorporate information (e.g., feedbacks with information on other users in similar situations). Other authors, like Fischer, address the topic of energy efficiency by studying the effectiveness of feedback techniques. Fischer reviews other studies and the factors they indicate as determinants of efficiency, he also conducts an analysis to find the factors of the highest importance [5]. Ayres et al. have also used feedback techniques to allow customers to learn how to reduce their electricity and natural gas consumption, so that public utilities can reduce energy consumption at low cost [6]. Suciu et al. in [7] propose a tool that estimates potential savings which can be achieved by adopting photovoltaic solutions in Small and Medium Enterprises (SMEs). Some proposals focus on the proper regulation of temperature, so that room temperature does not change drastically from one moment to another. When users make optimal decisions (efficiency measures) about the use of heating and air conditioning, they are able to reduce energy waste and lower the cost of bills. Among these measures we find, for example, the proper regulation of temperature, so that room temperature does not change drastically from one moment to another. An increase by one degree in the temperature implies a 7% increase in the consumption of energy. Temperature is regulated using the heat of the sun to heat the building or house and by airing the building when the outside temperature is greater, since this will lower the contrast between inside and outside temperature. An essential aspect is detecting the air leaks in the building where the heat and cold enter, causing more energy to be consumed. It is also important to know which areas of the building or flat are occupied and at what times, so that the heating can be switched off or lowered and programmed to be switched on when the person returns to the house. 

The authors of [8] proposed measures that allow one to make better use of energy in buildings and reduce annual gas bills by 70%. Public office buildings urgently require the implementation of energy saving solutions. This is because amount of energy they consume is much higher in comparison to other types of buildings and houses. The users of public buildings are often not careful about saving energy (switching lights on/off), as they do not have to pay the energy bill. Thus, it is necessary to propose effective measures that will change user habits, especially in public buildings [9]. Changing the user’s habits in their own home is not as difficult because whenever they make correct decisions (efficiency measures) about the use of heating and air conditioning, they reduce energy waste and lower the cost of their bills. 

From the above works we can see that it is necessary to identify the factors that contribute to increased energy consumption to be able to maintain the desired temperature without increasing its use while heating the building in winter or cooling it in summer. Some of these factors include the location of the building, the state of the building and climate conditions [10]. Although these factors predetermine the energy use to be greater, the behavior of the people who work in these building also has a major influence. However, it is not enough to discern the actors and factors that influence energy saving and energy efficiency. The saving potential is dependent on a wide range of factors, such as local stakeholders on the energy market, regulations, weather, environment, energy prices and availability or financing opportunities. Thus, what we need to do is to combine and improve previous approaches, with the aim of developing effective solutions that will help users save energy.

In the reviewed literature, we did not find any architecture that would collect all the necessary data and provide solutions to the problems that have been described in the previous paragraph. For this reason, this work proposes an energy efficiency model which allows one to save energy in homes and buildings through temperature management. Energetic efficiency is achieved in this architecture through a temperature monitoring approach in all the rooms, corridors and all the exterior walls of the building. Data from diverse types of sensors and third-party sources is used to provide additional information which is used by our optimization algorithm in the process of data analysis. This allow the system to recommend the best possible energy saving techniques in the building and to propose changes in the habits of the inhabitants [11]. Minor et al. present a CBR approach for inert energy management systems that aims to reduce energy wastage in overheating and over cooling for buildings [12].

The goal of the work presented in this article is to provide a new system for the analysis of the parameters that influence the consumption of energy. On the basis of this analysis the system will make decisions that will optimize the use of energy. This system has been developed using a multi-agent system as the base architecture for technical implementation. The information required for the analysis is obtained through the temperature and occupancy data provided by the sensors; this is possible thanks to the deployment of a WSN which facilitates communication between the different devices that make up the system. The communication between the hardware and software of the system (sensors, actuators, etc.) is performed by the agents. 

The main contributions of this paper are as follows: acquisition of information related to the inhabitants in a non-intrusive way thanks to the use of sensors or a CBR system, conjunction of the current indoor and outdoor temperature together with the future temperature to prevent temperature jumps of the HVAC system that make the energy consumption increase strongly. All this has allowed the development of a system that uses a combination of information from the habits and preferences of inhabitants and the variables that influence energy expenditure to achieve energy savings. 

The rest of the article is structured as follows: Section 2 describes diverse state of the art proposals in the area of temperature control systems and studies on behaviour analysis for this type of systems. We also look at the progress achieved in this area and outline the technologies employed by these systems. Section 3 provides a full description of the system proposed in this work, including the functionality of each of its components. Section 4 details the case study and discusses the results obtained through an empirical implementation in a real scenario; the proposed system is deployed in an office building and monitors the temperature of each room. Finally, Section 5 sets out the main conclusions drawn from this research.

## 2. Related Work

Nowadays, the need to carry out exhaustive temperature control in the home is very high, especially with the recent changes in energy certification requirements. It is crucial to understand how to design a system that achieves efficiency in energy consumption through the monitoring of parameters. With the aim of achieving this objective, the following section reviews the current state of the art in this area. 

### 2.1. Temperature Control Architectures

Different proposals have already approached the task of improving the efficiency of buildings by focusing on the points that cause inefficiency, such as temperature changes. One of the most commonly used solutions is the use of PID controller. The PID controller consists of three different parameters: proportional, integral and derivative. The proportional value depends on the current error. Integral depends on past errors and derivative is a prediction of future errors. The sum of these three actions is used to adjust the process of a control function such as the position of a control valve or the power supplied to a heater. Other techniques are used to modify the temperature parameter settings in the boiler or the air conditioner. Shein et al. presented a CPS-based HTC system design, consisting of a hybrid controller and a PID controller [13]. In this work, they monitored the desired temperature of a room at all times with an optimum resource cost using two actuators using a supervisory controller and a PI controller. In some works, neural networks have been used to adjust the PID controller in numerous processes. One of these processes is learning the inverse dynamics model for the control of temperature, which is then configured as a direct controller of the process. Khalid and Omatu demonstrated that the use of a neural network offers many benefits, in comparison to a conventional proportional-plus-integral (PI) controller [14]. Other works use diffuse logic along with neural networks to adjust the Heating, Ventilation and Air Conditioning (HVAC) temperature in an office space. A combined neuro-fuzzy model for the dynamic and automatic regulation of indoor temperature in a building, forecasts indoor temperature; these forecasts are used to feed a fuzzy logic control unit that simulates change in the temperature control system [15]. 

Temperature control systems have not only been based on PID controllers. Since the PID constants can sometimes be incorrect due to the lack of understanding of the temperature control process and the possible changes in its parameters, it has been decided to use other techniques that do not make the system as complex. For this reason, other types of control systems, like Programmable thermostats (PT), have been researched. PTs have also been used in order to obtain considerable energy savings, while maintaining comfort. In this field, Gao et al. [16] presented the concept of a self-programming thermostat which automatically creates an optimal setback time by detecting the occupancy statistics of a home. The system monitors occupancy using simple sensors in the home, similar to those already used in typical safety systems, and the user defines the desired balance between energy and comfort using a single, intuitive knob. Thanks to this system, Preliminary results obtained from using this system showed a reduction in the demand for heating and cooling, up to 15%, but they can reach higher percentages if the variables that influence energy consumption and comfort parameters of people living in rooms or buildings are monitored.

### 2.2. User Behaviour Systems

There have been several works focusing on the domotization of different indoor spaces, such as homes, offices or entire buildings [17,18]. Some of these studies examined the influence of the users’ behavior on the consumption of energy, however, these works are fairly simple due to the fact that at the time they were conducted the cost of domotizing a home was higher than at present. One of these works was conducted by Mozer, whose approach was to observe the lifestyle and desires of the inhabitants to allow the system to anticipate and adjust to the needs of the users while maintaining energy consumption low [19]. For this purpose, it is based on an RC thermal house model, with a neural network that learns deviations and the actual behavior of the user. Other studies have studied the behavior of users in the manual control of windows in office buildings [20]; this study, evaluated the behavior of users by simulating office occupation. 

However, only a few researches studied the parameters that influenced energy consumption out in a real environment. For example, the case study conducted by Hoes et al. [21]. They studied the parameters that influenced users to open windows, such as the season, outdoor and indoor temperature, time of the day, presence. In the energy balance of a building it is clear that the influence exerted by the user is determinant, some studies have performed simulations of user behavior to optimize the design phase of building [21]. The results were obtained with the tool developed in the study, they indicated that the behavior and presence of the user should be evaluated in detail in order to be able to optimize the design of the building for real users and their preferences. To manage energy efficiently, Bayesian networks were used to predict user behaviour from a multi-modal sensor [22]. Therefore, an automated system should be developed to autonomously obtain the variables that influence energy consumption, taking into account the presence of people in the building.

### 2.3. Acquisition, Analysis of Data

HVAC equipment requires a monitoring system through which it is possible to control and manage the temperature of each radiator and air conditioning system independently. For this purpose, it is necessary to collect data on the location of each radiator and air conditioning system. In the pursuit of greater efficiency, these control systems should be complemented with data, such as the indoor and outdoor temperature of each office [23]. In addition to including the sensors’ temperature data intended for this purpose, it is convenient for the system to feed on new data, such as the temperature forecast for the next days or weeks, this knowledge is used by the system to optimize temperature management. The gradual increase or decrease of temperature allows to save energy, since it prevents rapid temperature changes. There are several platforms such as OpenWeatherMap, or the State Meteorological Agency (AEMET) from which this information can be obtained through their APIs. The temperature data collected by the sensors, together with information on temperature forecasts, allows the system to manage the temperature on the basis of these parameters. However, the accuracy of management and thus, energy efficiency, can be improved by including information about the behavior of users, such as presence in the office or their working schedule. It is therefore essential to collect data from various situations and conditions, such as the presence of people in the building and the way HVAC devices are being used. It is crucial to have efficient sensor networks which collect indoor and outdoor temperature data. Sensors that detect the presence or absence of people are also necessary, since the data collected can later be converted into useful information. In this regard, a distributed sensor network is a requirement since it permits to obtain and analyze data obtained from offices, the main composition of these networks should include a motion sensor, an acoustic sensor, and presence sensors [24,25]. 

Moreover, it is so important to have a large-scale sensor network for detecting the presence of people in a building and to have the methods and techniques that allow us to analyze the data collected by it. In this regard, Dodier et al. developed a suitable analysis method to determine occupancy in commercial buildings by deploying a network of passive infrared occupancy sensors and the use of algorithms based on Bayesian networks and belief networks [24]. The sensor network in each office consisted of three PIR occupancy detectors and a sensor that detects when the phone was off-hook; these allowed to establish occupancy with certainty. The importance of this proposal lies in the application of a belief network paradigm to the problem of energy management. This construction model of sensor networks demonstrates useful inference for a control system with these characteristics. Another approach, which pursued the same objective as the previous work, is the one presented by Hailemariam et al. in which, thanks to the depletion of a heterogeneous matrix of passive infrared sensors, occupation could be determined in real-time [26]. In this work, decision trees were used to perform the classification and to explore the relationship between different types of sensors, the characteristics derived from sensor data and occupation. This work stands out since it achieves 98.4% accuracy, when applying a combination of decision trees and multiple motion sensor characteristics. From these works, we learn of the importance of using motion, acoustic and presence sensors for different data analysis techniques, however, the overflow of information can have unproductive results [26]. The data collected by the sensors can be converted into useful information by developing active occupation patterns that allow to simulate energy demand [27] and dynamic control systems [28], however, this gives us information about schedules real occupants of the offices that allow to simulate the behavior of the occupants and predict future consumption within the building. 

### 2.4. Proposal Using a CBR

In addition to exploring techniques for predicting the presence or absence of people at work, it is recommended to include a system that acquires knowledge about the work calendar. This provides the system with knowledge on the workers’ non-working periods or vacation. Also, the hours at which workers enter and go out of the office, as well as their meal times. This information helps the control system to adjust the temperature of radiators or air conditioning. Apart from previously described studies which simulated user behaviour to predict energy consumption [29,30], this information is normally not included in the energy management systems proposed in the literature. CBR methodology functions on the basis of prior knowledge; these systems can aid energy management systems in the decision-making process, by reusing previously retrieved cases. CBR systems review each new solution and once it is executed, the past case is retained in order to be reused in a possible future solution. The use of CBR brings with it many advantages; it applies expert knowledge, provides quick suggestions and decisions for a particular type of problem and stores solutions for future applications (incremental learning), unlike decision trees or ANN whose adaptation is not so rapid [31,32]. However, they may not provide valid or really adequate solutions if the stored case set is small [33]. In this type of problem is an effective measure once similar cases are available, the value of the variables and the decision taken. This decision is mainly based on the value of the variables, which describe each type of problem (in this case the variables to be considered would be calendar data, weather data, office data, social data, workers’ data, etc.), as was done in the following work [34]. 

The use of CBR systems in this problem is not new, it has already been used in studies such as the one proposed by Hong et al. where it acts as a support system in the decision-making process that works to reduce consumption of electrical energy in elementary school facilities [35]. The CBR algorithm makes decisions on the basis of past cases and its efficiency is improved with the use of algorithms such as GA, ANN and MRA. Another study in the literature used detailed electricity consumption measures: consumption of all the fans belonging to the cooling system, heating and indoor lighting [36]. This information is generally not available in most buildings, so new models were developed to predict this information using typically available measurements. Reference [36] compared the use of ANN and CBR in the exact prediction of energy loads in buildings. One of the great contributions of this study is the use of the PCA technique for the reduction of variables used in the decision-making process; it demonstrated that although the variables were reduced by the PCA, no significant differences in the performance of both models were produced and both became slightly more accurate when eliminating variables that add noise and variability. 

### 2.5. Standard Data Communication Protocol—BACnet

Existing commercial home automation systems seek to achieve savings in the objectives they pose, however, existing commercial systems do not provide a global vision that would allow to add new objectives to the ones already included in the system [37]. Since specific protocols are used in these systems, they are closed to the inclusion of new devices. New devices and machine that allow to monitor different types of variables and factors in the environment, are being developed by different manufacturers continuously. In order to be able to connect these devices and use them for a common purpose: communicate, send information and create universal systems, a suitable communication protocol is required.

Building Automation and Control Networks (BACNet) is a communication protocol whose development began in 1987. Its purpose is to standardize communications between building automation devices of different manufacturers, and to allow data to be shared and the equipment to work together easily. It is a standard protocol in the US and European markets and in more than 30 countries, besides it is an ISO global standard [38]. This set of rules for the creation of interoperable “GTC” networks, is used as a means of integrating air conditioning management systems (air conditioning, ventilation, radiators), integration with external systems (chiller’s, VRF, RCF, power counts, frequency drives and others) and/or lighting systems. BACnet is the standard protocol in the management of HVAC as it stands out for its scalability capacity between costs, performance and size of the system, also allowing to add new innovations and characteristics at any time. Remote web access control also saves the time and money of those who are responsible for monitoring [39].

### 2.6. Multi-Agent Systems

The characteristics of the agents that make up multi-agent systems allow this paradigm to be employed in a wide variety of research fields, such as data analysis in bioinformatics [40,41], image classification of facial faces according to gender and age [42] or WSA data fusion [43] but with a nexus that allows to work with, and analyze large amounts of data. A large amount of data allows us to obtain useful information in very diverse areas, and to recognize data trends (behavior patterns, consumption patterns, spending patterns) [44]. In addition, the autonomy with which multi-agent system (MAS) agents are provided, allows them to interact with each other without any human intervention. Their ability to perceive changes in the environment and react to them makes multi-agent systems an ideal approach for obtaining data from an environment and for responding to these changes with appropriate actions. Features such as extensibility and flexibility make it possible to add new functionalities or include other algorithms and sensors. These advantages allow us to adopt this approach in the problem of energy waste produced by changes made by users in temperature programming, as discussed by McArthur et al. [45,46].

Multi-agent systems are often applied to the field of building automation, due to their capacity to deal with more complex systems. Diverse HVAC process management systems have been developed by making use of the advantages provided by multi-agent systems, with the aim of optimizing energy consumption [47,48,49,50]. Wang et al. used a multi agent system to develop a control system with an intelligent optimizer. The proposed system allowed to manage energy and comfort in intelligent buildings, with the aim of achieving energy efficiency [51]. Looking at air conditioning management systems, the proposal of Cai et al. uses a multi-agent system for the management of buildings in centralized air conditioning systems [52]. In this work, the optimization problem was reformulated as several sub-problems, each being solved by an agent individually. Another study, proposed by Al-Daraiseh et al., leveraged multi-agent systems for energy optimization focused on predicting the shutdown and power-up time of the HVAC system in Higher Education Institutions: this system considered factors such as outdoor climatic conditions and the presence of people [53]. However, the proposed system lacked automation and the values had to be introduced manually being; what allowed to achieve even higher savings. The rest of the article describes the energy management system proposed in this article; it considers the advantages of the previous proposals in this field and tries to solve the deficiencies that these systems presented in terms of flexibility, automation and pattern learning.

## 3. System Overview

This section focuses on the technical details related to the system proposed in this work. It describes many different technical aspects, such as the collection of data with sensors, transmission and communication of data between the devices and the system, conversion of data into useful information, use of this information in the decision-making process which allows to achieve efficient use of energy.

### 3.1. Arquitecture

**Figure 1:**
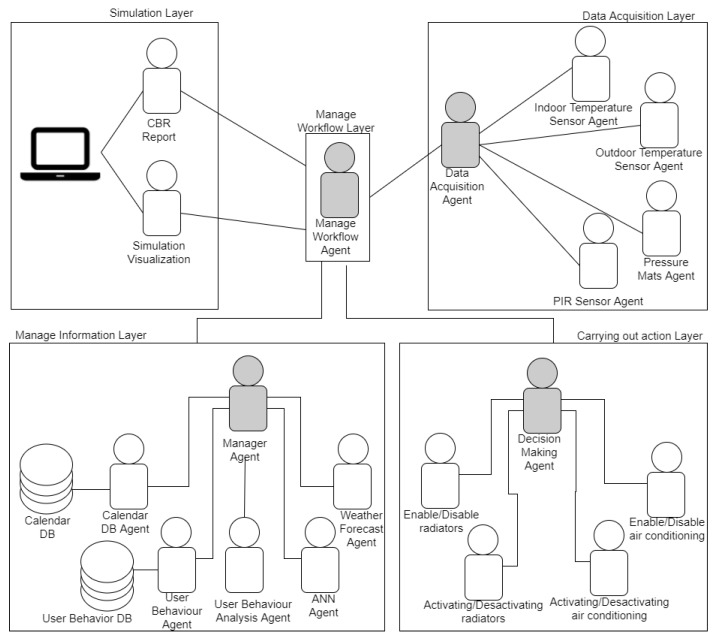
Multi-agent system arquitecture.

Multi-agent system agents use their communication capabilities to obtain the data collected by the sensors and IoT devices, destined for the collection of indoor and outdoor temperature and occupation data. This data is sent to other agents that perform analysis tasks. In turn, other agents obtain data from weather forecasts and analyze the behavior of the users [54,55] and even interact with the work calendar in order to know of the periods in which temperature should be reduced (in the absence of workers) and increased (prior to their return). These characteristics enable to program gradual changes in the temperature and do not allow abrupt changes in the temperature that cause high energy consumption. This is why the multi-agent system presented in this paper aims to optimally manage the temperature of an office building, including monitoring and real-time control of radiators or air conditioning. It also establishes temperature patterns that fit the energy efficiency frames and the comfort of the facility users. In order to fulfil this purpose, the multi-agent system is specifically designed to analyze sensor data and include data from external information sources [56]. The GAIA approach has been used in the development of the multi-agent system, since this methodology is focused on the analysis and design of software systems based on intelligent agents [57]. The system’s functionality is carried out by the agents in the system, where each agent is assigned a different role. The agents are grouped into five layers, according to the affinity of the activities each of them perform, as shown in Figure 1.

- *Data acquisition layer*: a layer that consists of four types of agents (indoor temperature agent, outdoor temperature agent and presence agent). The indoor and outdoor temperature agents collect temperature every 60 s. These agents are connected with the distributed sensors by means of the middleware. Knowledge about the presence of users in the offices is obtained through Passive Infrared Sensors (PIR). One of the problems that may arise is that if the employees are not seated in their work place, the PIR sensor will not detect their presence, however, although they are in the office. To counteract this problem, pressure mats have been placed at the entrance of each office, this allows to obtain knowledge on the number of people that enter and leave the office. Thus, even if the PIR sensors do not detect people in their work place, the number of people in the office is kept record of.
- *Information management layer*: which is responsible for carrying out data analysis processes, namely analyzing temperature, presence, calendar and user behaviour data, so that decisions can be taken subsequently. Specifically, the difference in the indoor temperature of the rooms that are exposed to the outside and those that are surrounded by the other rooms from all sides. The user behavior analysis agent analyzes the behavior patterns of the users in each room, by establishing a schedule for the times at which they usually leave and enter the room, periods of stay, if they are present on weekends. This information is stored in a database intended for this purpose. The weather forecast agent makes requests for the meteorological information to OpenWeatherMap (http://openweathermap.org/api), this information is used in the process of increasing or decreasing the temperature based on the forecast of temperatures for the following days. In this way, the system makes a gradual increase or decrease in temperature. The academic calendar agent communicates with the agent manager in order to inform him of vacation periods, thanks to this agent, the agents in the execution layer can take this information into account when making decisions.
- *Execution layer*: this layer makes decisions on the basis of the information received from the other agents that make up the system. The activate/deactivate air conditioning agent executes the action of turning the air conditioning on or off, the activate/deactivate radiators agent executes the action of turning the radiators on or off. The temperature increase/decrease of the radiators/air conditioning agent executes the action of increasing or decreasing temperature the radiators or the air conditioning in each of the rooms or in the common area, by some degrees. These actions are carried out through the communication between the agents and the Smart thermostat.
- *Workflow management layer*: which includes an agent in charge of the workflow of the rest of the layers. It is also responsible for establishing the correct order for the activity of each agent. The workflow analysis agent collects information about the settings and can repeat previously performed sequences for expression analysis. This aspect allows to automate repetitive analysis tasks. These workflows are stored in a database that is managed by the workflow management agent, which stores and retrieves the settings.
- *Simulation layer*: this layer manages the case-based reasoning system (CBR). The CBR performs tasks in four different steps, in order to obtain a workflow that adapts to the conditions of the presented problem. First, the multi-agent system retrieves the relevant cases in order to solve the problem using the workflow memory. Once a relevant case is recovered, this workflow is previously adapted to suit the new problem; to changes in temperature conditions, user presence, events in the academic calendar, etc., once adapted, it is reused in the new problem. Later, the review step is performed in order to avoid unwanted steps in the workflow for the following process: for the retaining of the solution in a database. The system first simulates the solutions provided by the CBR, in this way only the most relevant actions are chosen for the reduction of energy consumption and those that allow to obtain the greatest savings are chosen. This layer has an agent with the user allows to interact to know the consumption data, and see the data that are collected in real time by each sensor deployed.

An IoT architecture that obtains and analyzes personal data requires the incorporation of security procedures that keep the privacy of such data intact and prevent external vulnerabilities. For that reason, each part of the multi-agent architecture (devices, field gateway, cloud gateway and connections) presents different associated security problems. Authentication of the devices is done through transport layer security (TLS) and IPsec, also using the pre-shared key (PSK), TLS RSA/PSK, IPSec and RFC 4279 are used in the field gateway. To prevent traffic interception or interference of communication between the device and the gateway, traffic is encrypted using PSK/RSA.

### 3.2. Data Adquisition

This section details the way in which the system collects the occupancy data, indoor and outdoor temperature, and the forecast of climatic conditions.

#### 3.2.1. Obtaining Employee Presence Data

The sensing system is simple and allows to detect occupation and to collect values for the outside and inside temperature of the office. The prototypes are programming in C using Arduido IDE.

**Figure 2:**
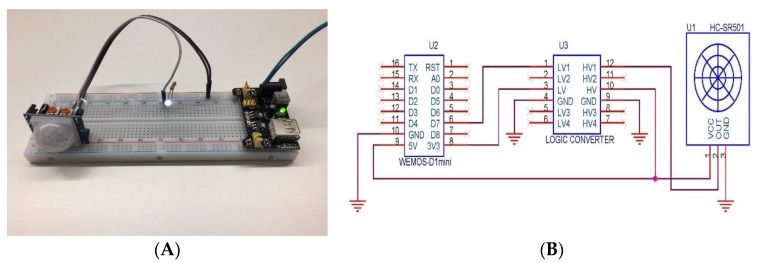
Presence detection system circuit. (**A**) shows the prototype and (**B**) shows the connections.

**Figure 3:**
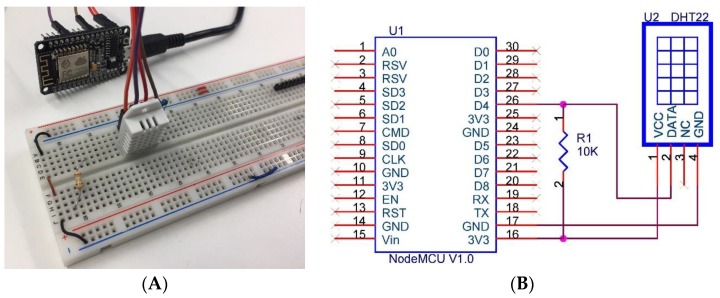
Prototype of the temperature acquisition system. (**A**) shows the prototype and (**B**) shows the connections.

- *PIR sensors*: Knowledge about the presence of users in the offices is obtained through the deployment of passive infrared sensors (PIRs). These sensors are called passive because, instead of emitting radiation, they receive it. They capture presence by detecting the difference between the heat emitted by the users and the space in which they are. Once deployed, these sensors need a period of adaptation, in which these processes, “get accustomed” to the infrared radiation of the environment. In Figure 2 the presence detection system to be deployed in the work area is displayed in front of each worker. This simple system consists of the PIR sensor, a led, a resistance of 100 Ω, and is powered with a signal of 3.3 V. To prevent false detections by solar rays or other light sources, the sensor incorporates a small plastic lens that acts as a special light filter to eliminate this possibility. Once a non-presence detection occurs, this signal is sent to the PIR sensor in the Data acquisition layer, so that the system knows that the worker is not located in the workplace. In this way, if the presence system does not detect any workers in the whole office, the system changes its behaviour when adjusting the temperature. PIR sensors will only provide information about the presence or absence of people in their workplace. In order to have accurate knowledge on the presence of people in the office, pressure mats are used; they keep track of the number of people who have entered the office. If the PIR sensors report that there are no people in their workplace, the data obtained by the pressure mats are used. These pressure mats count the entrance and the exit of people in the office and in this way the number of people in it is obtained.
- *Temperature sensors:* It is necessary for the system to the indoor temperature values of the offices since it allows it to incorporate this information into the temperature adjustment algorithm. This is done thanks to the DHT22 sensor that is used to obtain temperature and humidity values, this sensor can be seen in Figure 3. In cases where the offices are exposed to the outside, the system also needs to collect outdoor temperature. The importance of obtaining external temperature data lies in the ability to know how the heat is wasted or how the cold air enters form the outside, these data allow the system to regulate the temperature better. This sensor allows us to measure temperatures between −40 °C and 125 °C with an accuracy of 0.5 °C, humidity measurement between 0% and 100% with an accuracy of 2–5% and a sampling frequency of 0.5 Hz.

#### 3.2.2. Obtaining Temperature Data from Offices

**Figure 4:**
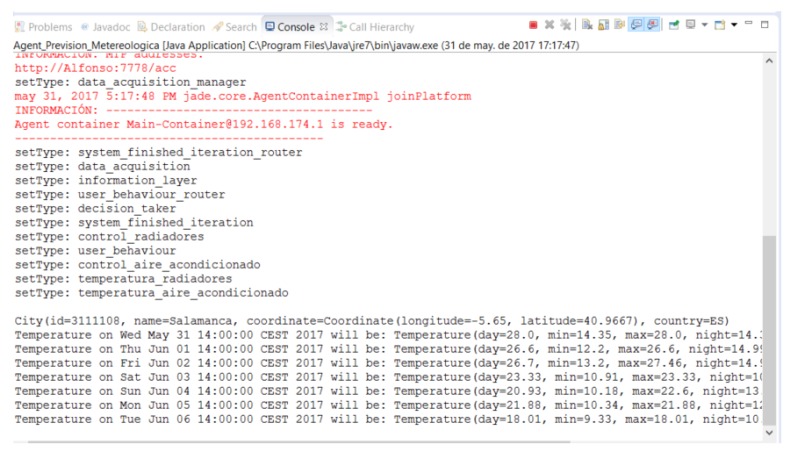
Weather forecast agent.

To obtain the meteorological forecast of the next days (a week or fortnight) meteorological information provided by the OpenWeatherMap API is used. OpenWeatherMap is a weather service based on the VANE Geospatial Data Science platform for the collection, processing and distribution of information about our planet through easy-to-use tools and APIs. In this regard, the architecture includes a Weather Forecast Agent who is responsible for obtaining the forecast information for the next week or fortnight (according to the configuration settings) by making requests to the OpenWeatherMap API. In Figure 4 we can observe the Weather Forecast Agent obtaining the minimum and maximum temperature of the day, the average temperature in the morning, in the afternoon and at night. These measurements are sent to the Manager Agent who will send the information to the Decision-Making Agent if the information has changed, or if the Calendar DB Agent has notified that the next week is festive and therefore the weather forecasts of no interest.

#### 3.2.3. Using Calendar Data to Make Decisions

The collection of data on the presence of employees in the workplace, the outdoor and indoor temperature, or weather forecasts, is necessary for the proper adjustment of temperature and the decisions that the system has to make. However, equally important to the acquisition of data is prior knowledge of whether there will be any human activity in the offices. The knowledge of whether the employees will be in the offices can be acquired thanks to the work calendar of the country, the region and the sector in which the employees in the office work. If there is a period of one week or a fortnight of inactivity, the system will lower the temperature or turn off various radiators or air conditioning in order to minimize energy consumption. However, the system will not turn everything off as it will then require more energy to reheat or cool the offices before workers come back; this would result in consumption being greater to what could have been saved.

### 3.3. Data Transmission

**Figure 5:**
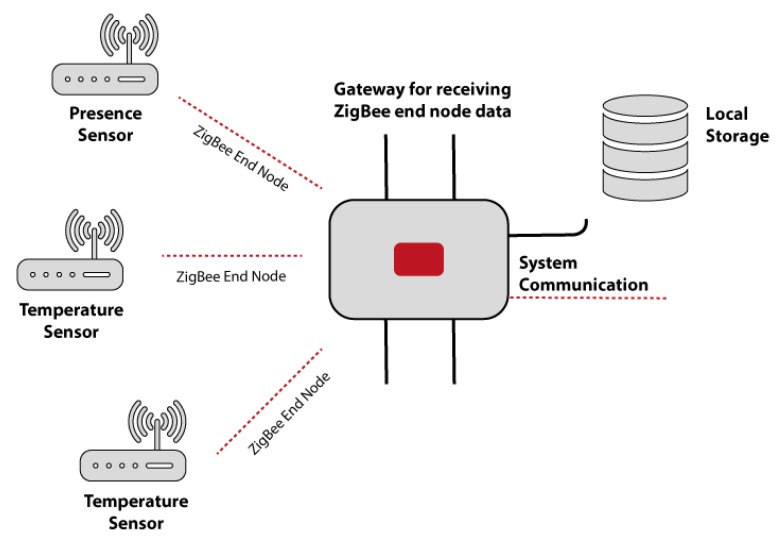
Transmission through the ZigBee protocol of the data collected by the sensors.

The data collected by the outdoor and indoor temperature sensors, PIR sensors and pressure mats are used by the agents in charge of each of these sensors send the recollected data. The multi-agent system architecture is open, this means that it is possible to include new technology (new sensors) in it, so in this regard the architecture has been provided with a standard data transmission. The use of this form of data transmission prevents us from having to develop a new functionality in the agents in charge of receiving information from the sensors. The communication protocol used by the architecture is BACnet [58]. The radiators, heating and air conditioning systems that are part of the system are compatible with this protocol, which allows for the individual or group control of these devices via WiFi or ZigBee connection [59]. When the data is sent by ZigBee there is a hub that collects and transmits the data to the multi-agent system as shown in Figure 5, unlike the data sent by the sensors that communicate via WiFi that are sent directly to the system by REST services. In this way, the communication of each device is replaced with a common set of communication rules which use a common language so that all devices communicate in the same way [60].

In the presented architecture, the enable/disable radiators agent, activate/deactivate radiators agent, enable/disable air conditioning agent and activate/deactivate air conditioning agent can communicate with the radiators, heating and air conditioning systems of the area through the Java distribution of this protocol (https://github.com/infiniteautomation/BACnet4J).

### 3.4. Learning Schedules Using a CBR

In this section, it is described how the proposed CBR framework was adopted to the present study, in order to predict the work schedule of the employees’ in each office. The use of a CBR is required for the process of learning schedules, once the cases have been constructed with the data collected during the baseline period. This automatic learning is necessary in order to store data about the habits of the employees in each office.

The system predicts the schedule of each office in a two-stage process. The first stage obtains the data collected by the installed presence sensors (form the time presence is detected in the offices, until the time no employee is present). In the second stage of prediction, a model of each worker’s schedule is made, generating the pattern of entry and exit of each worker into the office. Once the prevailing schedules for each office have been identified (i.e., a case has been built), the database that stores the CBR cases is constructed with the prevailing schedules of each office.

**Figure 6:**
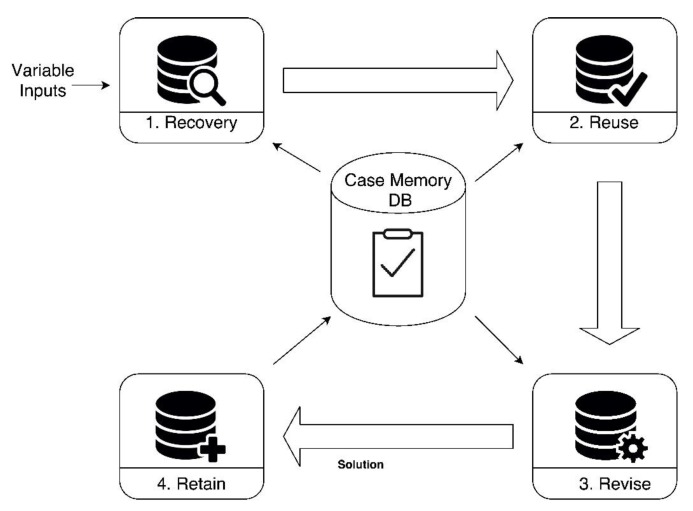
CBR agent for learning employee’s schedules.

To determine what the most influent parameters are, the correlation analysis and Kruskal–Wallis [61] methods were used. Kruskal–Wallis method is used to test if a dataset has originated from the same distribution, determining the dependency between the studied variable and the rest of variables. Once the most influent parameters are known, the estimation is carried out by the CBR agent as shown in Figure 6, which has previously trained a Multi-Layer Perceptron (MLP) where the inputs are timenow, timeback, timebackPeriod, timeleave, timeleavePeriod and timeperiodDay.

The input and output values of the neural network are rescued in the range [0.2, 0.8] since the selected activation function is sigmoidal and we do not want any extreme values in the training. In the middle layer 2*n* + 1 neurons are placed where *n* is the number of neurons in the input layer. This criterion is based on Kolmogorov’s theorem [62]. In the recovery phase, the system recovers the previously trained network associated with the structure of the corresponding greenhouse. In the adaptation phase, the network is used to generate the prediction. Finally, the data and training are updated in the revise phase, once the target temperature has been reached and the amount of energy required is known. The ANN is trained periodically both manually, by using a graphic user interface to facilitate the process, and automatically, when a defined number of new cases has been included in the system. In manual training, the evolution of the error is analyzed with a set of training data. When the error begins to reduce at a slower rate, the neuronal network is validated with the test data. The training continues, while the error produced in the data of the test reduces.

Once the schedule of the employees of each office participating in the case study has been estimated, the overall schedule must be assessed in order to be use by our optimization algorithm. The CBR system predicts the working hours of the employees of each office, so that the data collected from each employee of each office can be used by the in the optimization algorithm for energy consumption.

### 3.5. Energy Saving Algorithm

The developed savings algorithm is based on the analysis of the collected data, it considers the variables that are implied in the temperature changes in the offices. The algorithm has a dual functionality: conditioning (temperature adjustment) and decision making (turning off or on the air conditioning or individual radiators). Programmable thermostats can be used to define a fixed setback schedule that optimizes temperature, minimizing abrupt changes (temperature increase/decrease by several degrees in a short period of time) by means of temperature and occupancy values. Most programmable thermostats on the market provide four programmable parameters (comfort temperature, energy-saving temperature, energy-saving period, temperature setback) that create backoff periods. This algorithm uses the knowledge provided by the CBR to learn about when workers leave the office each day *time_leave_*, and the time that elapses before they return *time_leavePeriod_*, either on the same day (lunch period) or until the next day *time_back_*, also the *time_backPeriod_* that the workers spend in the office. The temperature parameters: indoor temperature *tmp_indoor_* and outdoor temperature *tmp_outdoor_*, and desired temperature *tmp_desired_* and forecast temperature *tmp_forecast_* variables are obtained by the sensors. Other parameters that are considered are: the status of the various air conditioners and radiators *device_status_*. During the adjustment period, the HVAC system will increase or decrease the temperature to the temperature desired by the office workers. In Table 1 the description of all the variables used in the energy saving algorithm is indicated. Algorithm 1 uses the information processed by the multi-agent system (Sensors information, timetables learned, work calendar and temperature prediction).
**Algorithm 1** Energy saving algorithm1: **procedure** energySaving(*period*)2:   **if**
*time_period_* == Academic ∧ presence == Yes **then**3:     *tmpnow* = *tmpdesired* − *tmpindoor*4:     **if**
*room_type_* == Private **then**5:        **if** ((*T_back_* − 60 min *× tmp_now_*
*≤ time*) ∧ *time_backPeriod_ >* 60 min) ∨ (*T_back_*
*≤ time* + 30 min ∧ *time_backPeriod_ >* 60 min) **then**6:          **if**
*tmp_now_ >* 0 **then**7:             **while**
*proxT_back_* − 60 min *× tmp_now_*
**do**8:                *thermostat_tmp_* + 1                        ▷ Increased temperature9:          **else if**
*tmp_now_ <* 0 **then**10:            **while**
*proxT_back_* − 60 min *× tmp_now_*
**do**11:               *thermostat_tmp_* − 1                        ▷ Decrease temperature12:        **else if**
*room_type_* == Common **then**13.          **if**
*time_periodDay_ ƒ* = Night **then**14:            *n* = *time_now_*15:            **if**
*time_periodDay_* == Morning **then**16:                **while**
*time_periodDay_* = Morning **do**17:                  *thermostats_tmp_* = $\sum_{i=7}^{n}\sqrt{tmp_{indoor}i}$
18:                  **if**
*tmp_desired_* = *thermostats_tmp_*
**then**19:                    break loop20:           **else if**
*time_periodDay_* == Afternoon **then**21:                **while**
*time_periodDay_* = Afternoon **do**22:                  *thermostats_tmp_* = $\sum_{i=15}^{n}\sqrt{tmp_{indoor}i}$
23:                  **if**
*tmp_desired_* = *thermostats_tmp_*
**then**24:                    break loop25:           **else if**
*time_periodDay_* == Evening **then**26:                **while**
*time_periodDay_* = Evening **do**27:                  *thermostats_tmp_* = $\sum_{i=21}^{n}\sqrt{tmp_{indoor}i}$
28:                  **if**
*tmp_desired_* = *thermostats_tmp_*
**then**29:                    break loop30:           **else**31:            *thermostats_status_* = Off32:        **else if**
*time_period_* == NonAcademic ∧ *time_period_ ≥* 10 days **then**33:          *thermostats_status_* = Off34:        *thermostats_status_* = Off35:   **else**36:        *thermostats_status_* = On

The use of the algorithm allows the CBR agent to learn behaviour patterns, along with the other variables. This results in energy savings thanks to the knowledge of the workers’ schedules in the different offices and learn schedules to program intelligent thermostats [63].

One of the innovations of this algorithm is that it uses the learning capacity of the agents in the architecture to learn about the workers’ schedules and behaviour; this allows for a more in-depth analysis of the data since it is done automatically. Before conducting an in-depth analysis, the system checks if the data collected are equal to those collected for previous analyses, if so, the corresponding action is carried out automatically; it also helps to reduces costs. Moreover, this learning ability has been employed in gene selection works with very good results [40].

## 4. Case-Study

In order to make a valid evaluation of the system and to demonstrate that it constitutes an advancement in energy saving that is not caused by a temporary change in the behavior of users; different control groups in the case study setting are chosen for validation. 

The International Performance Measurement and Verification Protocol (IPMVP), which is the basis of the common European ICT PSP Methodology for calculating energy savings in buildings, proposes four different procedures for settling in the non-intervention consumption [64,65]. The first method consists in isolating the key parameters, the second method is the isolation of all parameters, the third method is whole facility and finally the fourth method consists in calibrated simulation. Out of these four options only the third option is useful for measuring the utility of the proposed system, since it does not have to assume a constant energy demand, also the variation in demand can be modeled with precision. The following subsection details the setting in which the validation of the proposed system has been performed.

### 4.1. Experimental Set-Up

In order to verify the efficiency of the proposed system in the optimization of energy consumption, a monitoring and evaluation experiment has been designed, in it the dependent and independent variables have been designed and they will be used to evaluate the effects that the operation of the system produces in the building. The case study was divided in three phases and an office control group was chosen for the evaluation. This approach consists in monitoring the consumption in the offices before applying the system. In the case study both groups will be included; the offices that implement the system and those that don’t. Furthermore, in last phase of the case study the system will operate in both groups.

**Figure 7:**
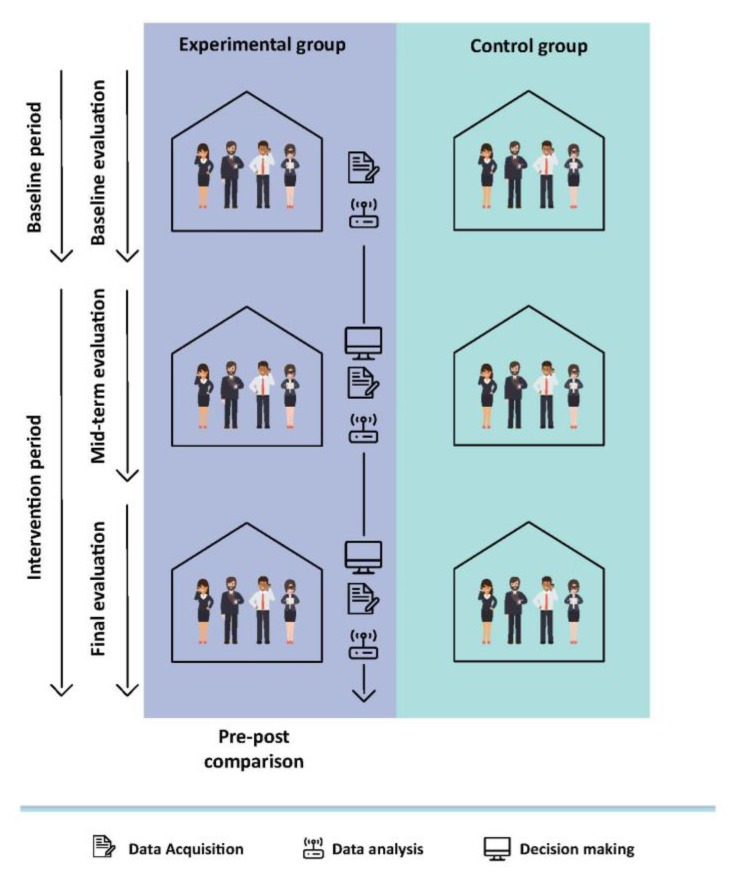
The case study population is divided into experimental and control group.

In these three stages, the variables that depend on energy savings (energy consumption, energy consumption behavior, awareness, demand peaks, on-site activity), and independent variables (price energy, perceived physical comfort, usability and utility) as shown in Figure 7. The dependent variables are the objective variables of the system; the system acts on them in order to obtain energy savings. Moreover, the independent variables are factors that may have an impact on the dependent variables. For the three steps described above, the dependent and independent variables will be collected. Thus, when the values of these variables were collected for both the experimental group and the control group in the same period, both groups experienced the same meteorological conditions, energy prices or holidays, in order to validate the results obtained.

**Figure 8:**
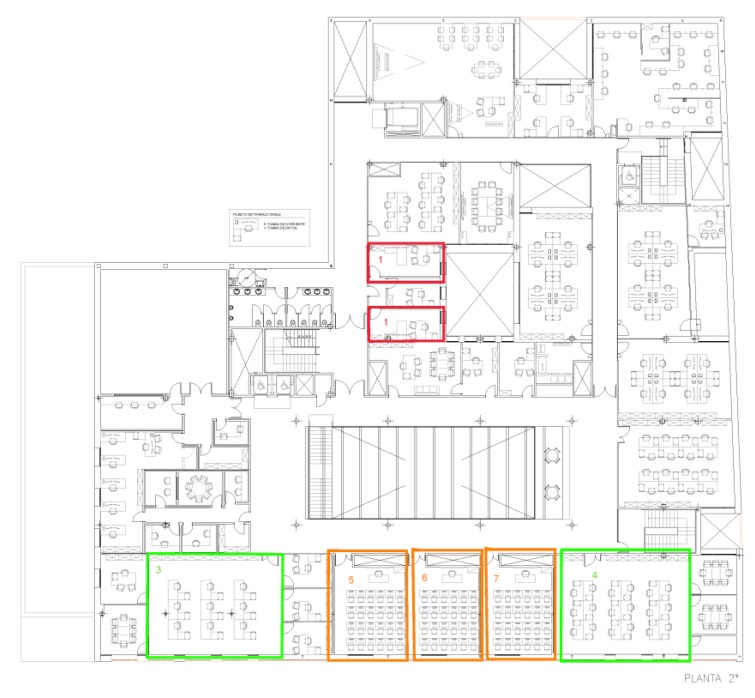
Situation of the offices in which the case study is carried out.

The setting in which the case study was carried out, consisted of seven offices which were positioned differently within the building (Edificio I+D+I, University of Salamanca), as shown in Figure 8.

These offices have the following characteristics: they are all located on the same floor in the building, they have a different cardinal point and their size is different, as shown in Table 2.

**Figure 9:**
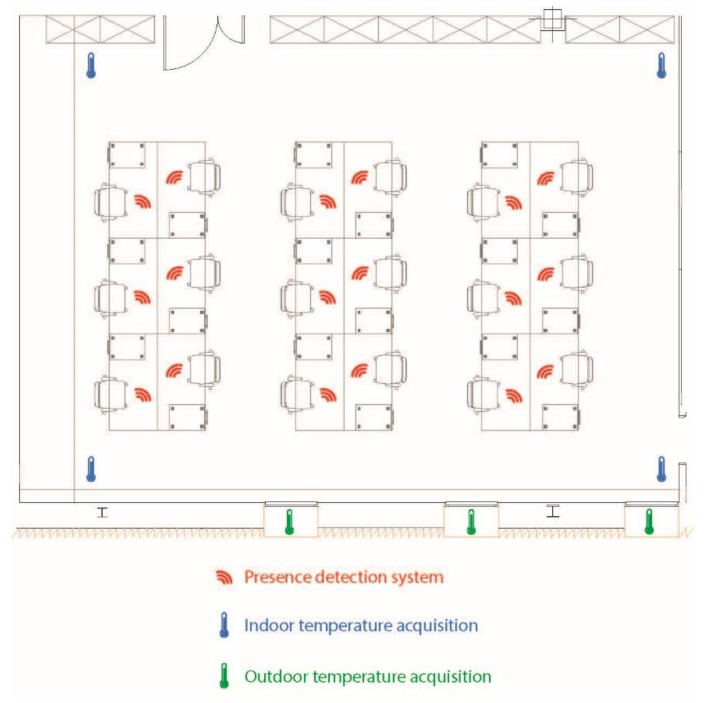
Example of the display of sensors in office 4.

The experiment is divided into three stages (baseline, mid-term evaluation, final evaluation) and each period lasts three weeks (from 9 January to 12 March), which allows for the correct evaluation of the system’s efficiency. The choice of the date in which the experiment was performed was due to the fact that in this period the number of feast days per month is similar and the maximum and minimum temperature did not vary as much from week to week. As described previously, in the baseline stage (9 January–29 January), only the case study group (offices 1, 4 and 5) and the control group (2, 3, 6 and 7) were monitored. In the mid-term evaluation stage (30 January–19 February), both groups were monitored and the system was already in operation in the offices belonging to the case study group. In the final evaluation stage (20 February–12 March) the system was operating in both in the case study group as well as the control group. In Figure 9 we can see how the sensors used for data collection are deployed in one of the offices. A presence sensor was deployed at each workstation, an external temperature sensor at each window and an indoor temperature sensor in each corner of the office.

### 4.2. Agents Coordination for Obtaining Energy Efficiency through the Communication of the Information 

**Figure 10:**
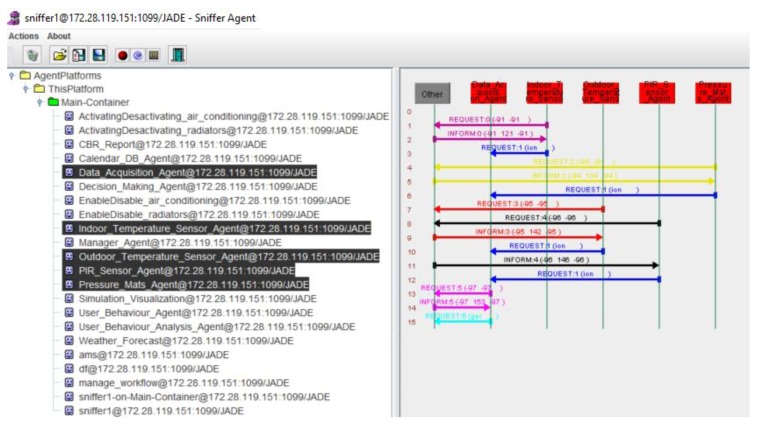
Communication between the agents of the data acquisition layer.

Thanks to the use of the multi-agent system, data from the indoor (*tmp_indoor_*), outdoor (*tmp_outdoor_*) temperature sensors, presence of workers (presence) in the case study offices were collected every 30 min. This information is managed by the data acquisition layer, as shown in Figure 10.

**Figure 11:**
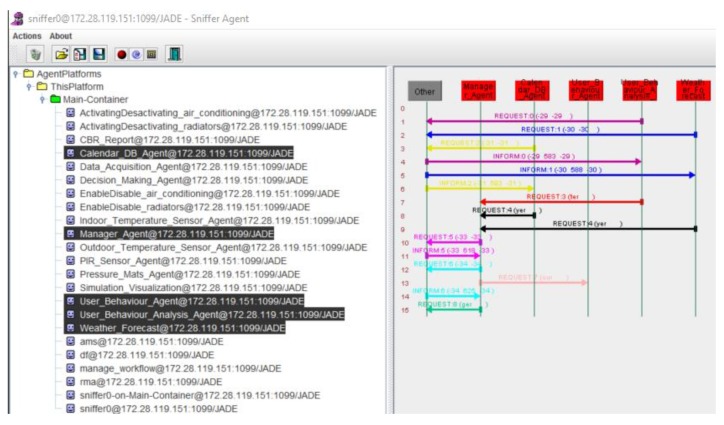
Communication between the agents of the information management layer.

The remaining information required by the algorithm is obtained by the agents that form the information management layer and through communication with the data acquisition layer. This information is related to the meteorological forecast or period type (*tmp_forecast_*, *time_period_*) and the other variables used by the algorithm, which are obtained by the CBR *(time_back_*, *time_backPeriod_*, *time_leave_*, *time_leavePeriod_*). The variables that are associated with the presence of employees in the offices are used by the user behavior analysis agent in order to store related data and generate more solutions for the optimization of energy consumption. In Figure 11 we can see the communication between the agents in the information management layer, in the process of obtaining the forecast data for the coming days, they obtain the behavior of the users (storage in the database of the CBR) and make an analysis of all the data.

The variables used in the analysis (*room_type_*, *thermostat_status_*, *thermostat_tmp_*, *time_now_*, *time_periodDay_*, *tmp_now_*, *tmp_desired_*) are obtained by the agents that are part of the execution layer.

### 4.3. Experimental Results

This section describes the results of implementing the system in a working environment (office building), since one of the objectives of the system is to be self-adaptive to the conditions and characteristics of any building. In order to verify that the system behaves effectively in terms of adaptability, it has been tested in an office building which has many different spaces and characteristics (location in the building, floor, geometric shape, dimensions, etc.) as described in the previous section. During the three weeks of the baseline period, the system has only collected data from the offices without taking any action. In the mid-term evaluation, the system has collected the values of all the variables, but only made decisions in offices 1, 4 and 5 and finally in the final evaluation (intervention period) the system already collected values and made decisions in all the offices, as seen in Table 3.

In Table 4 we can observe the consumption per stage (baseline and mid-term) of experimental and control group and the savings. In Table 5 we can see the results of the Student’s *t* test and the Levene test. The difference between the average baseline period and mid-term period is also significantly lower for the latter in all cases, with a *p*-value of 0.000 or close for all the different means. These results demonstrate the effectiveness of the algorithm in terms of energy savings.

**Figure 12:**
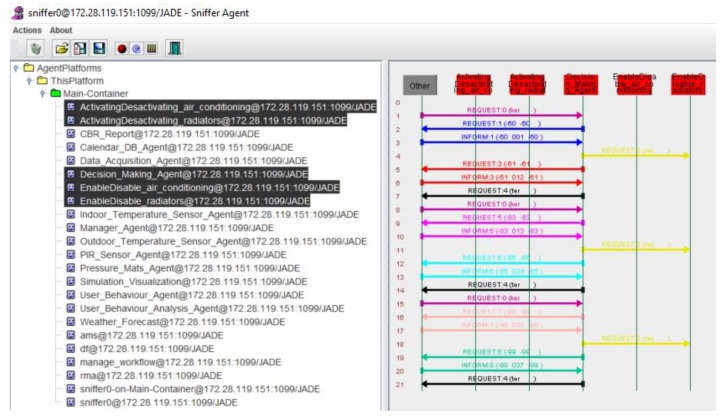
Communication between the agents in the decision-making process.

In Table 6 we can observe the consumption per stage (mid-term and final evaluation) of the experimental and control group and the savings. In Table 7 we can see the results of the Student’s *t* test and the Levene test. The difference between the average of the mid-term period and Final evaluation was also lower for the final evaluation period in all cases, with a *p*-value of 0.000 for all means. Standard deviations do not show significant differences between groups. This reinforces the result, showing that energy savings are achieved, as individuals within different groups tend to behave in the same way. These results reinforce the validity of applying a multi-agent system to collect consumption data and user behavior to obtain a behavior change that has an impact on energy savings by optimizing consumption. This decision-making process is done by communicating the agents of the carrying out action layer, as shown in Figure 12.

**Figure 13:**
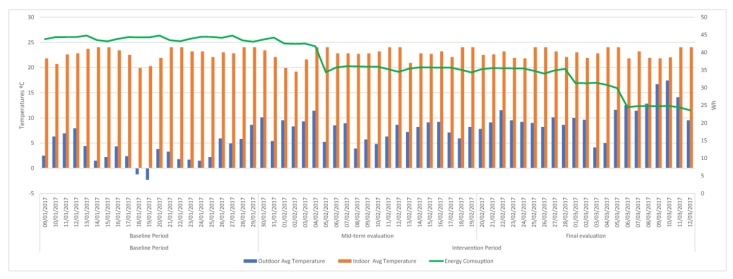
Energy consumption during the different periods of the experiments in office 4.

As shown in Figure 13 and Figure 16, in addition to monitoring the data during the two intervention periods, the system made its own decisions (programming the HVAC system temperature, switching on or off heating or cooling functions) energy consumption has followed a more stable linear trend. This indicates the stability of the temperature in the office and therefore that there are no sudden changes in energy consumption.

**Figure 14:**
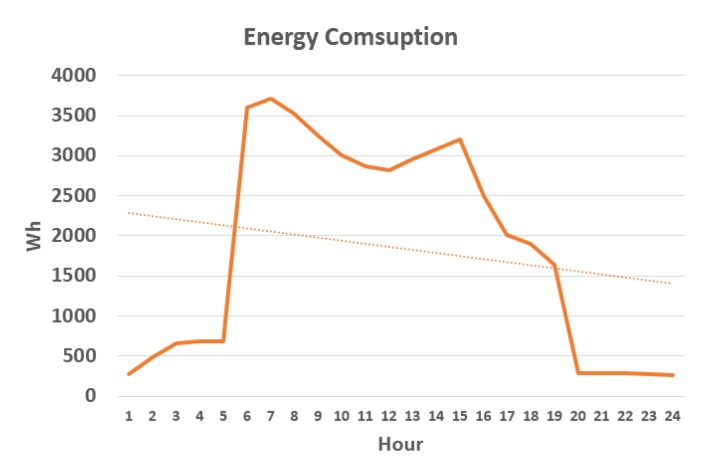
Energy consumption in office 4 of the first day of the mid-term evaluation.

**Figure 15:**
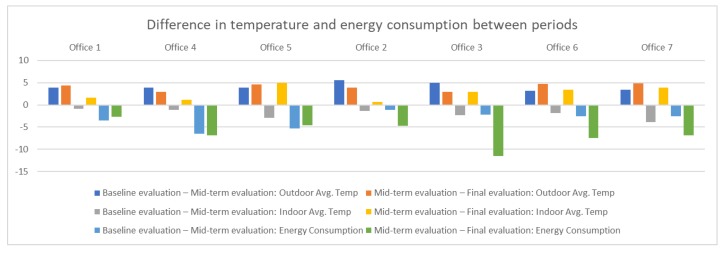
Representation of the difference between the temperature and the energy consumption between periods.

In Figure 14 we can confirm that the trend showing consumption is linear downwards from the start of the day (first day of the mid-term evaluation in office 4). In Table 8 we can observe the degrees of temperature that has increased or decreased and the increase or decrease of energy consumption, between the periods baseline evaluation and mid-term evaluation and mid-term evaluation and final evaluation. In the graph of Figure 15, the trend of temperature increase in the mid-term evaluation period with respect to baseline evaluation and of the final evaluation with regard to mid-term evaluation is perceived, this causes a lower energy consumption in all the offices, being clearly much less consumption between the offices of the experimental group than in the offices of the control group. This reduction in energy consumption in a different proportion in mid-term evaluation and in a greater proportion in the control group in the final evaluation period (similar to the experimental group) certifies the effectiveness of the system in the optimization of energy consumption.

**Figure 16:**
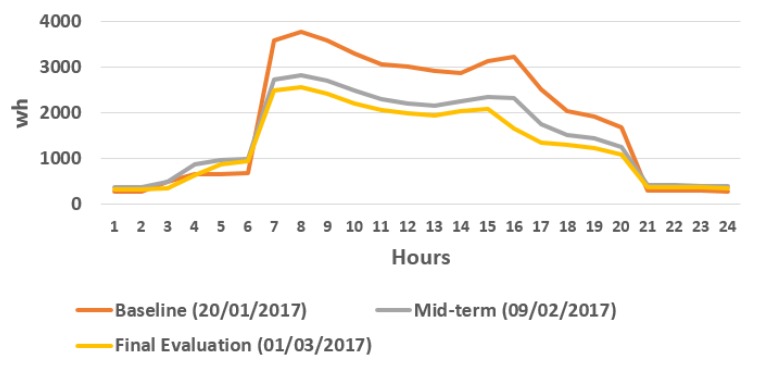
Office 4 energy consumption in Baseline Period, Mid-term evaluation and in Final evaluation.

Figure 16 shows how the consumption does not exceed 3000 kWh in Intervention period, being noticeable as it is even smaller in final evaluation due to the increase of the external temperatures.

It is clearer that an increase in the difference between outdoor and indoor temperatures increases energy consumption, and in the mid-term evaluation the consumption between offices is the same, and similar in final evaluation period (Table 9).

**Figure 17:**
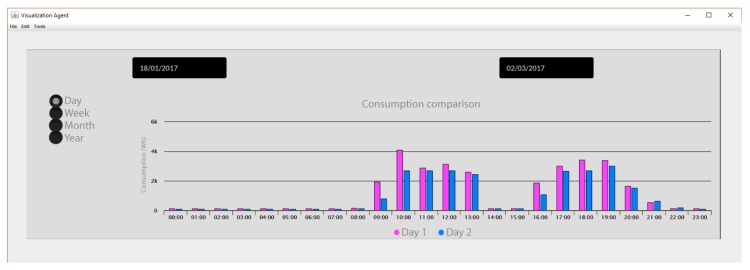
Energy consumption comparison at Office 4 by Visualization agent.

Apart from all the data that we can obtain thanks to the system with which we have generated graphs that allow us to accurately evaluate the operation of the system, through the visualization agent you can make comparisons about consumption in the different periods of which the experiment as shown in Figure 17.

**Figure 18:**
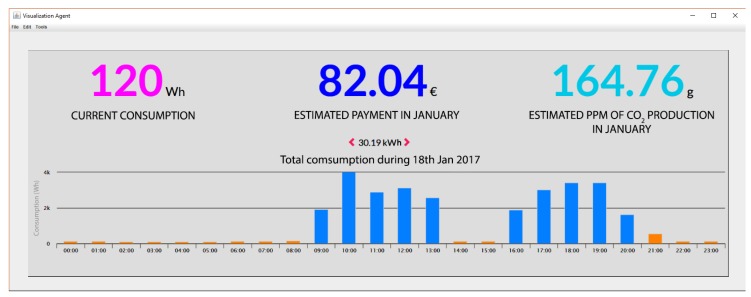
Details about consumption, invoice, CO_2_ production on a specific day.

It also allows to export all the data by means of diverse grouping methods (day, week, month, year) to show the current consumption, climatological details among others. In Figure 18 we can see the current consumption, the forecast of the invoice for that month, and the estimated CO_2_ production.

## 5. Conclusions

This paper has presented a novel multi-agent based approach for the optimization of energy consumption, caused by inefficient use of HVAC systems. The use of a multi-agent system in this energy management problem has been fundamental, since it allowed us to find out how users interacted with these devices and other external factors that had an influence on consumption. To get an understanding of the building’s internal and external climatic conditions, as well as office occupancy, the multi-agent system uses data from the sensors distributed in the offices. In addition, the system has knowledge of the weather forecast planned for the coming days, as well as the non-working or holiday periods; in this way, it is able to set the radiators and air conditioning optimally. The multi-agent system helped us to achieve energy savings in the office building by correctly programming the temperature of the radiators and air conditioning, by adjusting it to changes (radiators, air conditioning on/off) without making abrupt shifts; since this would increase the consumption of energy drastically.

A case-study in an office building during nine weeks has shown that the proposed solution achieves that the consumption of electrical energy is lower in the set of rooms managed by the multi-agent system. It can also be deduced that the decrease in the difference between outdoor and indoor temperature does not lead to a linear decrease in energy consumption. As can be seen in the results obtained, the algorithm developed for making decisions related to the on or off of the radiators or the air conditioning or the increase or decrease in the temperature of the radiators or the individual air conditioning accomplish an average energy savings of 41% in the rooms of an office building. Future lines of research will be concerned with incorporating agreement technologies in order that the optimisation is carried out taking into account all user comfort parameters.

## Figures and Tables

**Table 1:** Summary of algorithm variables.

Variable	Description	Units/Value
devicesstatus	Condition of radiators or individual systems	[On|Off]
presence	Presence of employees in the office	[Yes|No]
roomtype	Type of room	[Private|Common]
thermostatstatus	Thermostat status	[On|Off]
thermostattmp	Temperature at which the thermostat is programmed	°C
timenow	Current time	hh:mm:ss dd/MM/yyyy
timeback	Time the first employee returns	hh:mm:ss dd/MM/yyyy
timebackPeriod	Time the employee is in the office since entering	min
timeleave	Time the last employee leaves	hh:mm:ss dd/MM/yyyy
timeleavePeriod	Time the employee is out of the office since leaving	min
timeperiod	If today is an academic term or not	[Academic|NonAcademic]
timeperiodDay	Period of the day according to the hour	[Morning|Afternoon|Evening|Night]
tmpnow	Current temperature in the office	°C
tmpdesired	Desired average temperature by employees	°C
tmpforecast	Expected outdoor temperature	°C
tmpindoor	Indoor temperature	°C
tmpoutdoor	Outdoor temperature	°C

**Table 2:** Total consumption in kWh during the 30 days of baseline data collection, during the 30 days of the experiment, difference in the consumption and savings between the two periods.

Consumption	Office 1	Office 2	Office 3	Office 4	Office 5	Office 6	Office 7
**Plant Number**	2	2	2	2	2	2	2
**Room Type**	Private	Private	Private	Private	Private	Private	Private
**Cardinal Situation**	SO	SO	SE	SE	SE	SE	SE
**Office Dimensions**	14.29 m^2^	14.22 m^2^	88.77 m^2^	88.83 m^2^	43.43 m^2^	43.76 m^2^	43.47 m^2^
**Number of Workplaces**	3	3	9	18	25	25	25
**Number of Windows**	1	1	3	3	1	1	1
**m^2^ of Window Surface**	3 m^2^	3 m^2^	8.1 m^2^	8.1 m^2^	6.3 m^2^	6.3 m^2^	6.3 m^2^

**Table 3:** Average temperatures and energy consumption by period.

			Experimental Group			Control Group			
			Office 1	Office 4	Office 5	Office 2	Office 3	Office 6	Office 7
Baseline period	Baseline evaluation	Outdoor Avg. Temp (°C)	3.54	3.54	4.34	2.45	2.52	4.7	4.43
		Indoor Avg. Temp (°C)	21.68	22.75	22.60	22.65	22.29	22.66	23.71
		Energy Consumption (Wh)	15.06	44.03	26.1	15.04	44.00	25.8	25.7
Intervention period	Mid-term evaluation	Outdoor Avg. Temp (°C)	7.38	7.48	8.2	8.02	7.48	7.86	7.79
		Indoor Avg. Temp (°C)	20.81	21.64	19.69	21.25	19.98	20.82	19.86
		Energy Consumption (Wh)	11.61	37.49	20.76	13.93	41.75	23.21	23.16
	Final evaluation	Outdoor Avg. Temp	11.7	10.36	12.85	11.94	10.4	12.54	12.59
		Indoor Avg. Temp	22.47	22.84	24.61	21.97	22.92	24.22	23.8
		Energy Consumption (Wh)	8.91	30.6	16.21	9.22	30.3	15.78	16.34

**Table 4:** Total consumption in Wh during the 21 days of baseline period, during the 22 days of the experiment (Mid-term evaluation), difference in the consumption and savings between the two periods.

	Experimental Group			Control Group			
	Office 1	Office 4	Office 5	Office 2	Office 3	Office 6	Office 7
**Baseline period Consumption (Wh)**	15.06	44.03	26.1	15.04	44.00	25.8	25.7
**Intervention period (Mid-term evaluation) Consumption (Wh)**	11.61	37.49	20.76	13.93	41.5	23.21	23.16
**Difference (Wh)**	3.45	6.54	5.34	1.11	2.25	2.59	2.54
**Savings (%)**	22.91	14.85	23.37	7.38	5.23	10.08	9.88

**Table 5:** Result of the Student’s *t*-test and Levene’s test performed to assess the difference of means and variances between the baseline data and the mid-term period (Intervention period). All the offices present a lower percentage of energy usage after the experimentation.

	Baseline		Mid-Term					
	Mean	Std.	Mean	Std.	*t*	*p*-Value (2-Tailed)	F	*p*-Value
**Office 1**	15.0638	1.90999	11.6110	1.31663	6.821	0.000	1.072	0.307
**Office 2**	15.0929	1.51719	13.9343	1.21753	2.729	0.009	0.588	0.448
**Office 3**	44.0300	1.80667	41.753	3.47799	2.661	0.011	16.65	0.000
**Office 4**	44.0033	0.58033	37.4938	3.48009	8.455	0.000	46.798	0.000
**Office 5**	26.1452	1.85345	20.7633	1.50320	10.335	0.000	0.036	0.850
**Office 6**	25.7962	1.23490	23.0152	1.67739	6.118	0.000	1.711	0.198
**Office 7**	25.7057	1.18029	23.1571	1.51395	6.084	0.000	1.547	0.221

**Table 6:** Total consumption in Wh during the 22 days of Mid-term (Intervention period), during the 22 days of Final evaluation (Intervention period), difference in the consumption and savings between the two periods.

	Experimental Group			Control Group			
	Office 1	Office 4	Office 5	Office 2	Office 3	Office 6	Office 7
**Intervention period (Mid-term evaluation) Consumption (Wh)**	11.61	37.49	20.76	13.93	41.75	23.21	23.16
**Intervention period (Final evaluation) Consumption (Wh)**	8.91	30.6	16.21	9.22	30.3	15.78	16.34
**Difference (Wh)**	2.7	6.89	4.55	4.71	11.45	7.43	6.82
**Savings (%)**	23.26	18.38	20	33.81	27.43	32.01	29.45

**Table 7:** Results of the Student’s *t*-test and Levene’s test performed to assess the difference of means and variances between the Mid-term period data (Intervention period) and the Final evaluation eriod data (Intervention period) data collected during the experimentation. All the offices present a lower percentage of energy usage after the experimentation.

	Mid-Term		Final Evaluation					
	Mean	Std.	Mean	Std.	*t*	*p*-Value (2-Tailed)	F	*p*-Value
**Office 1**	11.6110	1.31663	8.9124	1.64012	5.880	0.000	0.570	0.455
**Office 2**	13.9343	1.21753	9.2210	1.56940	10.874	0.000	2.659	0.111
**Office 3**	41.7538	3.47799	30.3310	4.24288	9.541	0.000	0.750	0.392
**Office 4**	37.4938	3.48009	30.6000	4.74534	5.368	0.000	3.645	0.063
**Office 5**	20.7633	1.50320	16.2100	1.33173	10.390	0.000	0.031	0.861
**Office 6**	23.0152	1.67739	15.7833	1.41483	15.102	0.000	0.030	0.864
**Office 7**	23.1571	1.51395	16.3433	1.28648	15.717	0.000	0.746	0.393

**Table 8:** Differences in temperature and energy consumption between periods.

		Experimental Group			Control Group			
		Office 1	Office 4	Office 5	Office 2	Office 3	Office 6	Office 7
**Baseline evaluation—Mid-term evaluation**	**Outdoor Avg. Temp**	+3.84 °C	+3.94 °C	+3.86 °C	+5.57 °C	+4.96 °C	+3.16 °C	+3.36 °C
	**Indoor Avg. Temp**	−0.87 °C	−1.11 °C	−2.91 °C	−1.4 °C	−2.31 °C	−1.84 °C	−3.85 °C
	**Energy Consumption**	−3.45 Wh	−6.54 Wh	−5.34 Wh	−1.11 Wh	−2.25 Wh	−2.59 Wh	−2.54 Wh
**Mid-term evaluation—Final evaluation**	**Outdoor Avg. Temp**	+4.32 °C	+2.88 °C	+4.65 °C	+3.92 °C	+2.92 °C	+4.68 °C	+4.8 °C
	**Indoor Avg. Temp**	+1.66 °C	+1.2 °C	+4.92 °C	+0.72 °C	+2.94 °C	+3.4 °C	+3.94 °C
	**Energy Consumption**	−2.7 Wh	−6.89 Wh	−4.55 Wh	−4.71 Wh	−11.45 Wh	−7.43 Wh	−6.82 Wh

**Table 9:** Relation between outdoor and indoor temperature difference and energy consumption.

		Experimental Group			Control Group			
		Office 1	Office 4	Office 5	Office 2	Office 3	Office 6	Office 7
Baseline evaluation	Mean (Outdoor-Indoor Temp)	18.14	19.21	18.26	20.2	19.77	17.96	19.28
	Energy Consumption (Wh)	15.06	44.03	26.1	15.04	44.00	25.8	25.7
Mid-term evaluation	Mean (Outdoor-Indoor Temp)	13.43	14.16	11.49	13.23	12.5	12.96	12.07
	Energy Consumption (Wh)	11.61	37.49	20.76	13.93	41.75	12.96	23.16
Final evaluation	Mean (Outdoor-Indoor Temp)	10.77	12.48	11.76	10.03	12.52	23.21	11.21
	Energy Consumption (Wh)	8.91	30.6	16.21	9.22	30.3	15.78	16.34
